# The Control of Diphtheria

**Published:** 1922-01

**Authors:** 


### THE CONTROL OF DIPHTHERIA.

**By A MEDICAL OFFICER OF HEALTH.**

DIPHTHERIA is mainly a disease of children, but infection is not altogether uncommon among adults. In cases of moderate severity the acute illness lasts for a week or ten days, but convalescence is prolonged over many weeks. Whether the patient is nursed at home or sent to hospital, the cure of the disease involves the expenditure of money—on doctors, on nursing and on antitoxin. Several days of work may be lost by the immediate contacts, and it is a costly matter to disinfect the house and bedding. It is not too high an estimate to suppose that every case of diphtheria costs at least £10, either to the individual or to the ratepayer. Probably three times that figure is nearer the truth. To state in the terms of money the value to their parents or to the State of the children who died of this disease is neither possible nor decent. Not all the riches in the world can take the place of the dead boy. So with all this expensive sickness and with the heavy mortality from the disease, it is evident that we, as a people, pay very hardly year by year for the diphtheria that is endemic in this country.

The Ministry of Health publishes from time to time reports on public health and medical subjects, and it has recently issued a Report (No. 10 of this series) on Diphtheria. The Report deals mainly with the value of the Schick test in the determination of susceptibility to the disease; but the rather vexed question of the swabbing of contacts is also considered. It will suffice to say here that the Schick test, in the hands of those who are used to it, has long passed the experimental stage; and is, in America, for example, and in some places in this country, employed with success in determining which of the population is susceptible or immune to diphtheria infection.

Lately the practice of swabbing diphtheria contacts, and of isolating "carriers" or those with positive swabs, has caused sub-acute controversy. It has long been recognised by all competent medical officers of health that a large percentage of carriers are for the moment non-infectious (because their organisms are of a non-virulent type), and, that since they are surrounded by a large number of immune persons in the general population, they are unlikely to give rise to infection. The medical officer of health, however, who believes that it is his duty "to do everything in his power to prevent disease" will take no chances; and will not permit the "carrier" to circulate freely—because there is a risk, a very, very small risk, certainly, but still a risk, of spreading the infection. The swabbing of contacts, of doubtful cases, of all children with sore throats, of patients before discharge from an isolation hospital secures the cautious medical officer of health from this very slight risk. The whole question at issue is, whether it is worth the trouble. Is it worth the trouble and expense?

Swabbing costs money. Swabs have to be examined in some laboratory; and, whether this be done in a public health department or at another laboratory, expense undoubtedly is involved. Indeed, each swab taken and examined may cost as much as a halfpenny! In those districts where the medical officer of health has no laboratory, each swab may cost, perhaps, half a crown or more; but mercifully, year by year, those ill-managed districts are becoming extinct—indeed, nowadays, a public health district without a laboratory is like a country doctor without a motor car, very rare indeed! Yes, swabbing costs money; and it takes time; it has to be organised, and it is definitely a nuisance and a lot of trouble! And all for what? For doubtful benefit and to avoid a slight, an infinitely small risk. It is the old question over again, "Is the game worth the candle?" Those whose children have had diphtheria are competent properly to answer that question. Would one whose boy has died of diphtheria allow a known, so-called non-virulent and healthy "carrier" to play with his other boys? Would he not rather advocate the swabbing of contacts and the isolation of the bacteriological carrier?

Medical officers of health are not looking about for more work. They have more than enough already. Very likely, most of the decent ones are doing a 70-hours week, and are not being paid for overtime. And yet they go on swabbing; and, in spite of the fact that the risk of letting loose the carriers is so very slight, they still insist on their isolation. And it may be for this reason—that virulence and non-virulence is not a fixed, unalterable entity, but, rather, is influenced by the passing of time.

A Klebs-Loeffler bacillus has been discovered in the nose of a perfectly healthy child. The bacteriologist says that this organism is non-virulent. It causes no harm to the child, and none to his intimates. The child is a carrier of a non-virulent bacillus. But it is known that bacteria (and protozoa also) undergo a slow process of evolution. Moore denies that. Bacteria, and the Klebs-Loeffler bacilli, multiply rapidly. They pass through many generations in a few hours, and it is reasonable to believe that they pass through many biological and immunological changes during the course of a few days or weeks or months or years. The influence of the host on an invading organism cannot be disregarded; and it would be very interesting if we could trace the non-virulent strain of the diphtheria bacillus until the time that it succeeds in killing the only son. If it could talk to us it would tell us something like this: "I lived in the throat of a boy at school. He was one of those immune kids, and I had a rotten time there. But at last he sucked his pencil; and I got on that, and went to the boy in the row behind, who had broken the point of his pencil and hadn't got a knife. He put me in his mouth and I found my way on to his tonsils—but he was one of those immune people—no fun at all. Still it bucked me up a bit, the change of climate. He took me, and my numerous family, home with him. Some of my children went to his father and mother, and a few more went to his brothers and sisters—five of them in all; but we have no luck in our family. Most of those children were

**The Control of Diphtheria—(*cont.*).**

as immune as their brother ; but one big girl certainly did get a bit of a sore throat. She wasn't ill with it. She had a young man, and my very great-grand-daughter made his acquaintance. He was one of those half-and-half immunity people, and our family did very well on his tonsils. They lived there for quite a long time. They gave his mother a bad sore throat, and she coughed at some of her youngsters, one of whom got a beautiful running nose. He went to school with it—a bad cold was what it was called ; and, would you believe me, they had no less than six cases of diphtheria in that school, until some horrid nurse came along with things they call swabs."

The truth of it all is that in the laboratory and in the test tube we can decrease and increase the virulence of an organism as we will. We, who happen to be bacteriologists, can do these things; but, at the same time, and most erroneously, some of us deny that this alteration of virulence can take place in the human test tube, or by human transference. Frequent and repeated animal passage, through monkeys or man, heightens the virulence of an organism. If the chain is broken at any link, if the non-virulent organism is killed or is allowed to die out at any period before it is stimulated into becoming virulent, then money and lives will be saved. So the diphtheria " carrier " will very likely do no harm at all—until his bacilli and their descendants have passed through many generations of insusceptible and susceptible hosts ; but after that you find—well, the diphtheria with a 10 per cent. mortality, the horror and the sorrow and the death.

### WORK FOR THE NEW LOCAL COMMITTEES.

### SIR NAPIER BURNETT'S PROPOSALS.

**(From a Correspondent.)**

IT is to be hoped that the Report on the financial position of the voluntary hospitals in England and Wales, issued last month by the Joint Council of the Order of St. John and the British Red Cross, has been well digested by hospital managers. Its authorship attracted public attention to the principal facts and figures, but we wish to remark now on certain minor but instructive matters which Sir Napier Burnett had the good sense to embody in it. In reference, for instance, to the article appearing last month, entitled " The Local Committee and Leicester," it is interesting to note what Sir Napier wrote with regard to the relation between county and Cottage Hospitals. He observed that the waiting lists pressed most severely upon the large hospitals, and declared that since the Cottage Hospitals are not always full a system of co-ordination is urgently needed to link the two types of Institution. Sir Napier suggested, as did our correspondent in the article mentioned, that the new Local Committees should arrange this co-ordination. In connection with Cottage Hospitals the point is made that they maintain a better average than the bigger institutions in the matter of cost per bed, and are said, in the mass, to show this year a surplus of £6 a bed. This statement is worth noting, for, in the future, if the best is to be made of the voluntary system, the condition of the Cottage Hospitals will require more attention, for they are essential to the well-being of every group to which they belong. We also learn from the Report that the payments of patients in Cottage Hospitals show a higher percentage, while the larger hospitals are said to gain only 6 per cent. of their income from this source.

In regard to workmen's contributions it is suggested that the Local Hospital Committees should seize the opportunity awaiting them and encourage the more systematic organisation of workmen's contributions. At present we learn that while some hospitals receive £114 per bed from this source others obtain £2 per bed only. It is manifest, therefore, that, notwithstanding the advantage possessed by hospitals in industrial areas in this respect, the present disparity argues lack of system generally.

Appended to the analysis of figures, is Sir Napier Burnett's reiterated plea for the general adoption of a simplified system of hospital accounts. At present the uniform system, as we have often pointed out, is disabled by the difference of dates on which the financial year is based.

In regard to hospitals with medical schools, Sir Napier insists upon their educational importance, and inquires whether the financial burden imposed by this work, which involves buildings, lecture rooms, cloak rooms, test rooms, elaborate scientific apparatus (all dead weight capital expenditure) should not entitle them to a grant from such a body as the University Grants Commission.

All these points raise another of great importance, namely, the tendency for the voluntary hospitals to restrict, or abandon the treatment of the sick poor, or to confine their relief to patients able to contribute. It is to the Local Committees that we must look for assistance in co-ordinating the over-filled voluntary hospitals with the often empty or unfilled beds in the Poor Law Infirmary.

---

LORD CAVE, whose Report on the Voluntary Hospitals led to the present scheme for assisting the hospitals, has agreed to serve on the Local Voluntary Hospital Committee for Surrey as one of the members nominated by the Hospital Commission. Considerable progress has now been made in the establishment of Local Voluntary Hospital Committees in various parts of the country, and the Commission has arranged for preliminary meetings of the Local Committees for Cornwall, Derbyshire, Glasgow, Monmouthshire, Salop, Staffordshire, Surrey, Warwick and Worcestershire.

